# Characterization of intestinal microbiota in alcoholic patients with and without alcoholic hepatitis or chronic alcoholic pancreatitis

**DOI:** 10.1038/s41598-018-23146-3

**Published:** 2018-03-19

**Authors:** Dragos Ciocan, Vinciane Rebours, Cosmin Sebastian Voican, Laura Wrzosek, Virginie Puchois, Anne-Marie Cassard, Gabriel Perlemuter

**Affiliations:** 1 INSERM UMRS U996 - Inflammation, Cytokines and Immunopathology, DHU Hepatinov, F-92140 Clamart, France; 2 Univ Paris-Sud/Paris Saclay, F-92140 Clamart, France; 3 INSERM UMR 1149, DHU UNITY, CRI, University Paris 7, Paris, France; 4 AP-HP, Pancreatology Department, Beaujon Hospital, Clichy, France; 5 AP-HP, Hepatogastroenterology and Nutrition, Hôpital Antoine-Béclère, Clamart, France

## Abstract

Excessive alcohol consumption leads to severe alcoholic hepatitis (sAH) or chronic alcoholic pancreatitis (CAP) only in a subset of patients. We aimed to characterize the intestinal microbiota profiles of alcoholic patients according to the presence and nature of the complications observed: sAH or CAP. Eighty two alcoholic patients were included according to their complications: CAP (*N* = 24), sAH (*N* = 13) or no complications (alcoholic controls, AC, *N* = 45). We analyzed the intestinal microbiota by high-throughput sequencing. Bacterial diversity was lower in patients with CAP, who had a global intestinal microbiota composition different from that of AC. The intestinal microbiota composition of these two groups differed for 17 genera, eight of which were more frequent in patients with CAP (e.g. *Klebsiella, Enterococcus* and *Sphingomonas*). There was no significant difference in bacterial diversity between the sAH and CAP groups. However, 16 taxa were more frequent in sAH patients, and 10 were more frequent in CAP patients. After adjustment for confounding factors sAH patients were found to have higher levels of *Haemophilus*. For alcoholic patients, specific intestinal microbiota signatures are associated with different complications. Patients with CAP and sAH also display specific dysbiosis relative to AC.

## Introduction

Chronic alcohol consumption is the major cause of pancreatitis and liver disease^1^. There is currently no available treatment, other than weaning off alcohol, for alcoholic pancreatitis or alcoholic hepatitis (AH).

The factors determining susceptibility to alcohol toxicity in a given individual remain unclear. Genetic variability and environmental exposure to various factors have been shown to play a role in the tissue predilection of alcohol toxicity^2,3^, but these factors explain only a fraction of the cases observed, suggesting the likely involvement of other factors.

The intestinal microbiota was recently identified as a major factor in AH^4^, and we have shown that individual susceptibility to the development of AH depends on intestinal microbiota profile in an animal model of AH^5,6^. Moreover, alcohol intake induces dysbiosis, triggering intestinal inflammation^7^. An increase in intestinal permeability, leading to a high load of pro-inflammatory bacterial products in the blood and the portal vein, is frequently observed in alcoholics and in animal models of AH, and these features contribute to liver inflammation and disease progression^8–11^.

Limited data are available concerning the intestinal microbiota in chronic alcoholic pancreatitis (CAP). A recent study reported a decrease in *Faecalibacterium prausnitzii* and *Ruminococcus bromii* levels in patients with chronic pancreatitis^12^. It has also been suggested that the oral microbiota may play a role in pancreatic cancer and chronic pancreatitis (CP), consistent with an impact of the microbiota on the pancreas^13,14^. Nevertheless, no data concerning intestinal microbiota modifications in patients with alcoholic pancreatitis have been published. Moreover, despite similar levels of exposure to alcohol, the specific consequences for the intestinal microbiota of AH and CAP are unknown.

In this study, our aim was to investigate the intestinal microbiota profile of alcoholic patients according to the presence and nature of the complications observed: AH or CAP.

## Results

### Clinical characteristics of the study population

Eighty-two patients were recruited between September 2008 and July 2016. The patients’ characteristics are summarized in Table 1. Patients with sAH had higher body mass index (BMI), a lower quantity of alcohol intake but for longer durations as compared to CAP and AC. Proton pump inhibitors intake was lower in patients with CAP as compared to AC and sAH patients.

**Table 1:** Clinical characteristic of patients.

	Chronic alcoholic pancreatitis (CAP) n = 24	Alcoholic controls (AC) n = 45	Severe alcoholic hepatitis (sAH) n = 13
Age (years)	51.5 ± 9.9	51.1 ± 8.5	54.1 ± 10
Sex (male/female)	21/3	41/4	11/2
BMI (kg/m^2^)	22.3 ± 3.4	23.9 ± 3.6	27.1 ± 6.6*
Alcohol intake (g/day)	143.8 ± 90.9	183.2 ± 113.1	95 ± 34.8*
Alcohol time (years)	13.1 ± 3.9	16.0 ± 10.5	22.1 ± 9.4*
Smoking (%)	22 (92)	39 (87)	10 (77)
Type 2 diabetes (%)	10 (42)	3 (7)**	2 (15)
Proton pump inhibitors use (%)	10 (42)	3 (7)***	5 (38.5)
CRP (mg/L)	37.4 ± 73.7	10.1 ± 11.5*	36.5 ± 33.3
AST (IU/L)	38 ± 35.1	67 ± 53.2***	296 ± 710.6***
ALT (IU/L)	44 ± 44.9	53 ± 42.0	84 ± 124.8
Total bilirubin (µmol/L)	25.0 ± 69.4	13.5 ± 5.4**	231.6 ± 209.2***
GGT (IU/L)	183 ± 294.1	303 ± 377.6	319 ± 231.9**
Glycemia (mmol/l)	7.3 ± 1.9	5.4 ± 0.9***	5.7 ± 1.4*
Serum albumin (mg/dL)	31 ± 7.9	38.1 ± 3.7***	27.1 ± 3.6
Platelets (× 10^9^/L)	324 ± 131.8	207 ± 84.7***	117 ± 110.0***
Prothrombin time (%)	93 ± 14.1	95 ± 7.5	34 ± 9.9***
Cirrhosis (%)	0	0	13 (100)
Liver biopsy (%)			13 (100)
Maddrey Score			57.5 ± 19.2
MELD			26 ± 7
Exocrine pancreatic insufficiency (%)	10 (42)	0	0

The data are expressed as the mean ± SD for continuous variables and *n* (%) for discrete variables. Comparisons between CAP patients and AC, and between CAP and sAH patients in Mann-Whitney tests or independent *t*-tests for continuous data and χ² tests or Fisher’s exact tests for discrete data. *p < 0.05; **p < 0.01; ***p < 0.001. BMI, body mass index; AST, alanine aminotransferase; ALT, aspartate aminotransferase; GGT, gammaglutamyltransferase; CRP, C-reactive protein.

### Intestinal microbiota profile in the study population

#### Intra-individual bacterial diversity (alpha diversity) according to the complication

**Figure 1:**
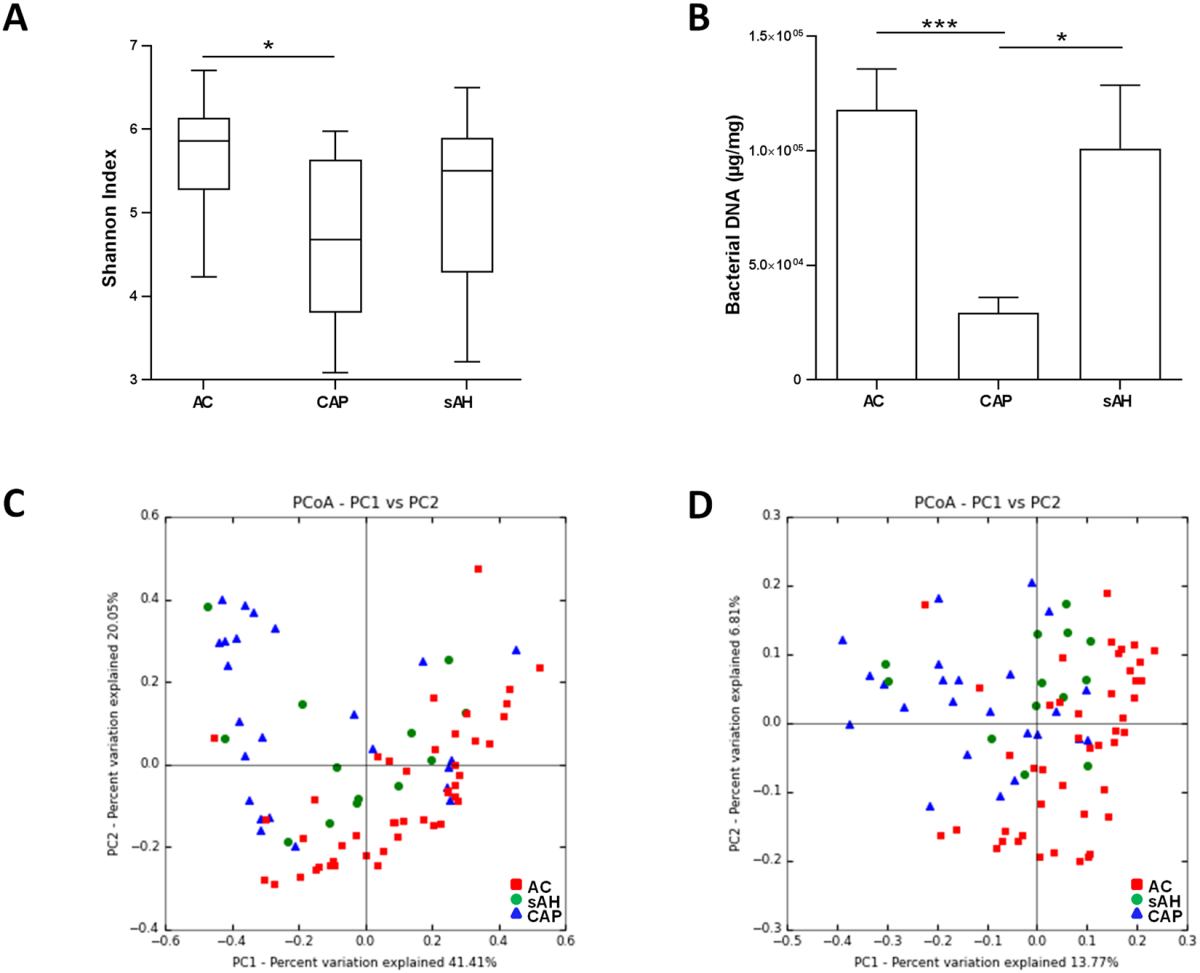
Intestinal microbiota profile in alcoholic patients without complications (AC) and with chronic alcoholic pancreatitis (CAP) or severe alcoholic hepatitis (sAH). (**A**) Box plots showing differences in microbiota alpha diversity based on the Shannon Index. (**B**) Concentration of total bacterial DNA in feces, and 16S rRNA DNA concentration (µg total DNA/mg; qPCR). *P*-values were calculated using a nonparametric ANOVA-test and Dunn post-hoc test for multiple comparisons. The rarefaction depth was 7,000. *p < 0.05, ***p < 0.001. (**C**) Principal coordinate analysis of the weighted UNIFRAC (p = 0.001) and (**D**) Unweighted UNIFRAC (p = 0.001) distances of the three groups.

Patients with CAP had a lower alpha diversity than AC suggesting a lower richness and evenness in the intestinal microbiota (Fig. 1A). This decrease in intestinal microbiota richness was also associated with a decrease in the amount of bacteria in feces, as shown by the quantification of bacterial DNA in stools (Fig. 1B). There was no difference in the alpha-diversity between CAP and sAH patients or between sAH and AC patients.

#### Inter-individual diversity in the intestinal microbiota of the study population (beta diversity) according to the complication

We compared the global composition of the intestinal microbiota in the study population according to the complication by calculating UniFrac distances and comparing them between the three groups. Principal coordinate analysis (PCoA) of weighted (Fig. 1C) and unweighted (Fig. 1D) distances identified three clusters, corresponding to the three groups of patients (unweighted, *p* = 0.001 and weighted, *p* = 0.001, respectively). These results suggest that the intestinal microbiota has a different composition (unweighted) and structure (weighted) in our study population. Therefore we performed comparisons between each group (CAP vs. AC, CAP vs. sAH and sAH vs. AC) in order to investigate the specific differences between our study population.

### Bacterial dysbiosis in patients with chronic alcoholic pancreatitis

#### Inter-individual diversity in patients with chronic alcoholic pancreatitis and alcoholic controls (beta diversity)

**Figure 2:**
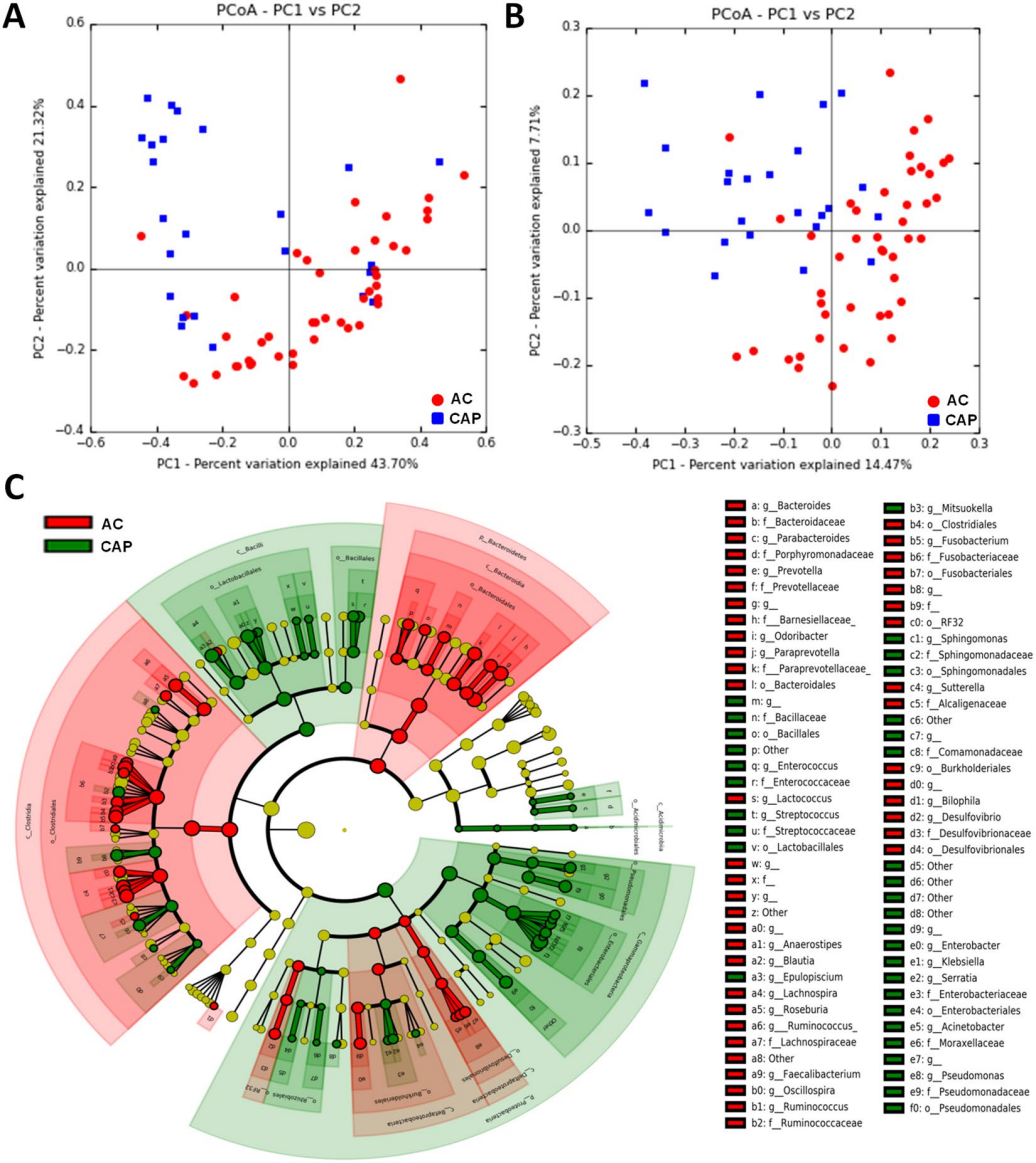
Intestinal microbiota analysis in patients with chronic alcoholic pancreatitis (CAP) and alcoholic controls (AC). (**A**) Weighted UniFrac distances (quantitative method reflecting differences in the structure of the intestinal microbiota between the two groups) and (**B**) Unweighted UniFrac distances (qualitative method reflecting differences in the composition of the intestinal microbiota). Each point represents a subject and the distance between the points is proportional to the similarity in the intestinal microbiota. The distances between groups are significantly different (*P*-value < 0.050 in the ANOSIM test). (**C**) Cladogram showing the taxa with the largest differences in relative abundance between CAP (green) and AC (red) patients. Circle sizes in the cladogram plot are proportional to bacterial abundance. From inside to outside, the circles represent phylum, class, order, family and genus. Only taxa with a LDA score >2 and a *p* < 0.05 in the Wilcoxon signed rank test are shown.

When comparing the global composition of the intestinal microbiota in CAP and AC patients, principal coordinate analysis (PCoA) of weighted (Fig. 2A) and unweighted (Fig. 2B) distances identified two clusters, corresponding to the two groups of patients (unweighted, *p corr* = 0.003 and weighted, *p corr* = 0.003, respectively). These results suggest that the intestinal microbiota has a different composition (unweighted) and structure (weighted) in CAP patients and AC.

#### Bacterial taxonomic differences between chronic alcoholic pancreatitis patients and alcoholic controls

We then investigated the specific differences in the intestinal microbiota at different taxonomic levels in these two groups. At phylum level, Proteobacteria were found to be more abundant in CAP patients than in AC (*p corr* = 0.02), whereas Bacteroidetes and Fusobacteria were less abundant (*p corr* = 0.006 and 0.03) (Supplementary Fig. 1). Fifty genera were found to differ in abundance between the two groups (*p corr* < 0.2) (Table 2 and Supplementary Fig. 1), and 43 of these genera were confirmed to be discriminant in LEfSe analysis (Table 2). Cladogram and LDA analyses identified the taxa specifically associated with CAP (Fig. 2C). After adjustment for sex, age, BMI, alcohol intake, smoking status, diabetes and proton-pump inhibitors use (using MaAsLin), 17 genera were found to differ in abundance between the two groups, with *Klebsiella, Enterococcus and Sphingomonas* specifically overrepresented in CAP patients (Table 2).

**Table 2:** Differences in the intestinal microbota between chronic alcoholic pancreatitis (CAP) and alcoholic controls (AC) at the genus level.

OTU			Increased in	Fold increase	Relative abundance		Mann-Whitney (ACP vs AC)		LEfSe (ACP vs AC)		MaAsLin (ACP vs AC)**	
Phyla	Family	*Genus*			ACP	AC	p-value	p corr	LDA	p-value	p-value	p corr
**Proteobacteria**	**Enterobacteriaceae**	Klebsiella[_s_pneumoniae]*	**ACP**	**309.38**	**0.00098**	**0.00000**	**0.00**	**0.00**	**2.94**	**0.00**	**0.00**	**0.02**
		Other[Klebsiella pneumoniae]*	**ACP**	**40.59**	**0.00735**	**0.00018**	**0.00**	**0.00**	**2.91**	**0.00**	**0.00**	**0.00**
		[Klebsiella//Enterobacter]*	**ACP**	**10.31**	**0.18392**	**0.01783**	**0.00**	**0.00**	**4.22**	**0.00**	**0.00**	**0.01**
		*Serratia*	ACP	27.50	0.00052	0.00002	0.00	0.00	3.25	0.00		
		*Enterobacter*	ACP	7.50	0.00136	0.00018	0.01	0.03	2.88	0.01		
		*Morganella*	ACP	19.64	0.00020	0	0.05	0.10				
	**Other**	Other[Klebsiella pneumoniae]*	**ACP**	**124.64**	**0.01583**	**0.00013**	**0.00**	**0.00**	**3.18**	**0.00**	**0.00**	**0.00**
	Pseudomonadaceae	*Pseudomonas*	ACP	364.29	0.00364	0	0.00	0.00	3.04	0.00		
		[Pseudomonas]*	ACP	47.02	0.00047	0	0.00	0.00	2.96	0.00		
	Moraxellaceae	*Acinetobacter*	ACP	1336.90	0.01337	0	0.00	0.00	3.14	0.00		
	**o__RF32**	***g_***	**AC**	**110.51**	**0.00003**	**0.00329**	**0.00**	**0.00**			**0.00**	**0.01**
	**Sphingomonadaceae**	***Sphingomonas***	**ACP**	**16.88**	**0.00027**	**0.00002**	**0.00**	**0.00**	**3.16**	**0.00**	**0.00**	**0.03**
	**Alcaligenaceae**	***Sutterella***	**AC**	**4.23**	**0.00139**	**0.00586**	**0.00**	**0.00**	**2.69**	**0.00**	**0.00**	**0.01**
	**Comamonadaceae**	[Aquabacterium parvum]*	**ACP**	**65.00**	**0.00062**	**0.00001**	**0.00**	**0.00**	**2.95**	**0.00**	**0.00**	**0.00**
		*Other*	ACP	7.21	0.00030	0.00004	0.02	0.04	3.24	0.02		
	**Desulfovibrionaceae**	***Bilophila***	**AC**	**10.16**	**0.00013**	**0.00133**	**0.00**	**0.00**	**2.73**	**0.00**	**0.00**	**0.00**
		*Desulfovibrio*	AC	2.93	0.00082	0.00241	0.02	0.05	2.37	0.02		
		***g_***	**AC**	**34.60**	**0**	**0.00035**	**0.00**	**0.00**	**3.25**	**0.00**	**0.00**	**0.01**
**Firmicutes**	**Enterococcaceae**	***Enterococcus***	**ACP**	**244.52**	**0.07530**	**0.00031**	**0.00**	**0.00**	**3.88**	**0.00**	**0.00**	**0.00**
		Other[Enterococcus]*	ACP	30.95	0.00031	0	0.01	0.02	3.20	0.01		
	**Bacillaceae**	[Enterococcus]*	**ACP**	**175.98**	**0.00391**	**0.00002**	**0.00**	**0.00**	**2.68**	**0.00**	**0.00**	**0.04**
		*Anoxybacillus*	ACP	277.38	0.00277	0	0.05	0.10				
	**Streptococcaceae**	***Lactococcus***	**AC**	**5.35**	**0.00018**	**0.00096**	**0.00**	**0.00**	**2.80**	**0.00**	**0.00**	**0.05**
		*Streptococcus*	ACP	2.69	0.07349	0.02736	0.01	0.02	3.65	0.01		
	Staphylococcaceae	*Staphylococcus*	ACP	21.43	0.00021	0	0.05	0.10				
	**Lachnospiraceae**	***Anaerostipes***	**AC**	**34.17**	**0.00009**	**0.00305**	**0.00**	**0.00**	**2.72**	**0.00**	**0.00**	**0.00**
		*Lachnospira*	AC	16.82	0.00049	0.00831	0.01	0.02	3.00	0.00		
		***Roseburia***	**AC**	**3.77**	**0.00502**	**0.01890**	**0.00**	**0.00**	**3.15**	**0.00**	**0.00**	**0.01**
		*g_*	AC	2.29	0.04609	0.10538	0.00	0.00	3.77	0.00		
		*Epulopiscium*	ACP	458.33	0.00458	0	0.00	0.00	3.01	0.00		
		*[Ruminococcus]*	AC	1.74	0.03011	0.05233	0.03	0.07	3.33	0.03		
		*Blautia*	AC	1.48	0.04202	0.06237	0.04	0.10	3.38	0.04		
		*Other*	AC	1.72	0.00480	0.00828	0.02	0.04	2.52	0.02		
	Ruminococcaceae	*Ruminococcus*	AC	6.88	0.00940	0.06469	0.00	0.00	3.74	0.00		
		*Oscillospira*	AC	1.86	0.00512	0.00950	0.00	0.01	2.71	0.00		
		*Faecalibacterium*	AC	2.27	0.01797	0.04075	0.01	0.03	3.40	0.01		
		Other[Ruminococcus albus]*	AC	11.02	0.00064	0.00702	0.00	0.01	2.83	0.01		
	Christensenellaceae	*g_*	AC	4.65	0.00041	0.00191	0.00	0.00	2.65	0.00		
	Veillonellaceae	*Dialister*	AC	6.04	0.00046	0.00277	0.06	0.11				
		*Mitsuokella*	ACP	1749.38	0.00555	0	0.01	0.02	2.98	0.01		
	Clostridiaceae	*Clostridium*	ACP	1.11	0.00395	0.00356	0.08	0.14				
	o__Clostridiales	*g_*	AC	1.62	0.03023	0.04885	0.03	0.06	3.28	0.03		
**Bacteroidetes**	**Bacteroidaceae**	***Bacteroides***	**AC**	**2.01**	**0.10708**	**0.21556**	**0.00**	**0.00**	**4.01**	**0.00**	**0.00**	**0.04**
	Prevotellaceae	*Prevotella*	AC	3.28	0.01673	0.05487	0.00	0.01	3.61	0.00		
	Porphyromonadaceae	*Parabacteroides*	AC	1.33	0.02304	0.03072	0.03	0.06	2.88	0.03		
	[Barnesiellaceae]	*g_*	AC	1.62	0.00467	0.00754	0.03	0.06	2.43	0.03		
	[Odoribacteraceae]	*Odoribacter*	AC	1.42	0.00276	0.00391	0.05	0.10	2.50	0.05		
	**[Paraprevotellaceae]**	***Paraprevotella***	**AC**	**38.07**	**0.00013**	**0.00476**	**0.00**	**0.00**	**2.86**	**0.00**	**0.00**	**0.00**
	[Paraprevotellaceae]	*g_*	AC	3.34	0.00009	0.00030	0.09	0.17				
**Fusobacteria**	Fusobacteriaceae	*Fusobacterium*	AC	2.73	0.00114	0.00312	0.02	0.05	2.46	0.02		

*OTU identified using BLASTN program (vBLAST + 2.6.0) from NCBI Blast against the NCBI 16 s Microbial database. Only hits with an overall sequence identity of 97% or more were considered.

**Covariates: age, sex, alcohol intake, smoking status, BMI, diabetes and proton pump inhibitors use.

ACP: alcoholic chronic pancreatitis, AC: alcoholic controls, LEfSe: LDA Effect Size, MaAsLin: Multivariate Association with Linear Models. In bold taxa with a p corr < 0.2 in MaAsLin.

### Differences in the intestinal microbiota between patients with chronic alcoholic pancreatitis and severe alcoholic hepatitis

#### Inter-individual diversity in chronic alcoholic pancreatitis and severe alcoholic hepatitis (beta diversity)

**Figure 3:**
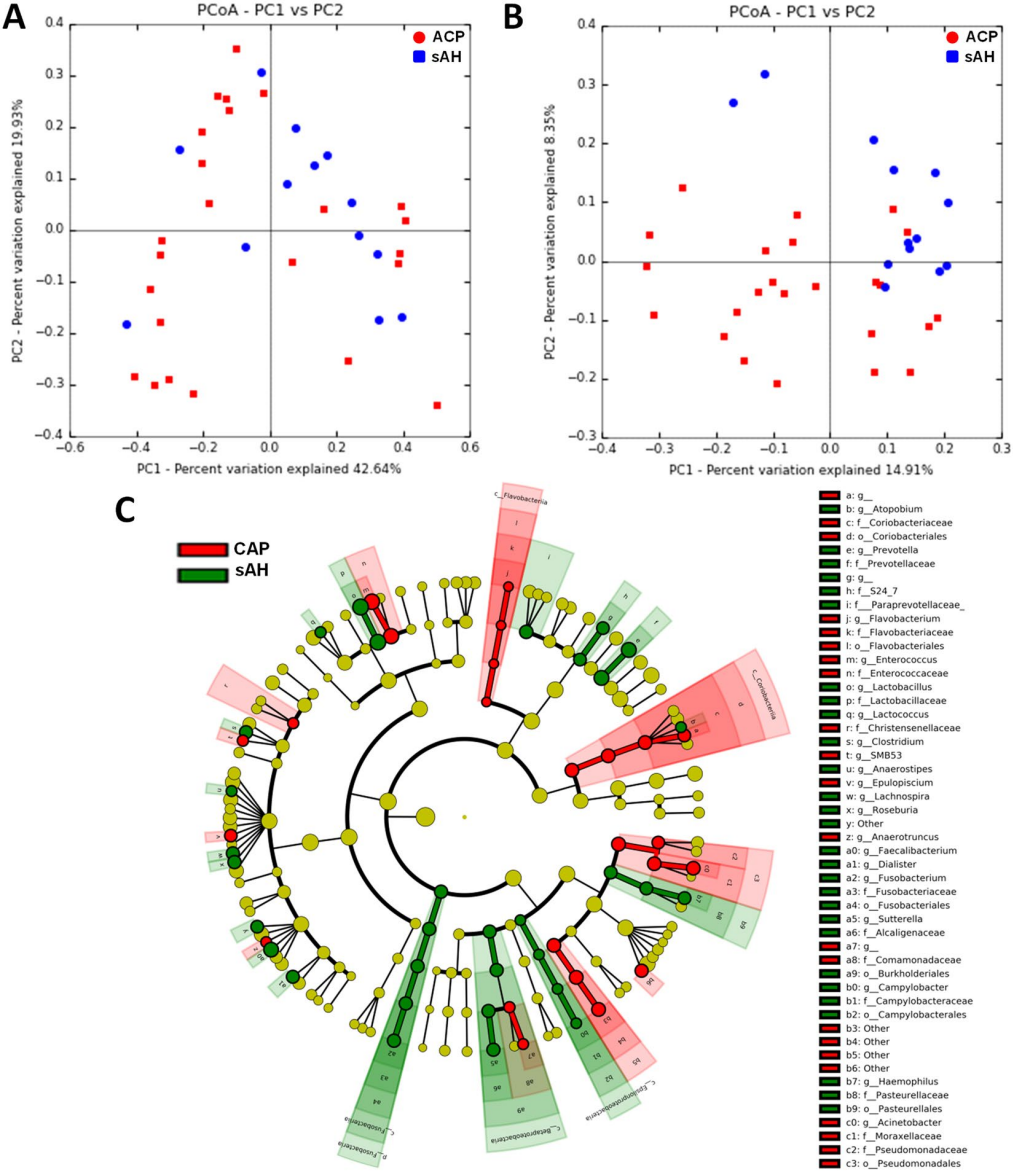
Intestinal microbiota analysis in patients with chronic alcoholic pancreatitis (CAP) and severe alcoholic hepatitis (sAH). (**A**) Weighted UniFrac distances (quantitative method) showing no difference in the structure of the intestinal microbiota between the two groups (*p* > 0.050); (**B**) Unweighted UniFrac distances (qualitative method), showing no difference in the composition of the intestinal microbiota between patients with chronic alcoholic pancreatitis (red) and patients with severe alcoholic hepatitis (blue), (*p* > 0.050). Each point represents a subject and the distance between the points is proportional to the similarity in their intestinal microbiota. (**C**) Cladogram showing the taxa with the largest differences in relative abundance between CAP (red) and sAH (green) patients. Circle sizes in the cladogram plot are proportional to bacterial abundance. From inside to outside, the circles represent phylum, class, order, family and genus. Only taxa with a LDA score > 2 and a *p* < 0.05 in the Wilcoxon signed rank test are shown.

Weighted distances did not differ between the two groups (*p corr* = 0.23, Fig. 3A). However, there was a trend towards two different clusters based on unweighted distances (*p*  corr = 0.07, Fig. 3B). These results indicate an absence of difference in the relative abundances of specific OTUs between CAP and sAH patients, but suggest that CAP and sAH patients may differ in terms of the presence/absence of some OTUs.

#### Bacterial taxonomic differences between chronic alcoholic pancreatitis and severe alcoholic hepatitis

No differences in the intestinal microbiota bacterial composition at phylum level were found between CAP and sAH patients (Supplementary Fig. 1). We found differences in abundance between CAP and sAH patients for 29 genera (*p* < 0.05, p corr < 0.20) (Table 3). For these genera, the abundance of *Haemophilus, Sutterella, Campylobacter, Lactobacillus, Faecalibacterium, Prevotella, Paraprevotella* and *Fusobacterium* was higher in sAH patients and that of *Serratia, Acinetobacter, Pseudomonas* and *Enterococcus* was higher in CAP patients. LEfSe analysis showed differences between the two groups for 26 taxa (10 being more abundant in CAP and 16 in sAH, Fig. 3C and Table 3). However, after adjustment for sex, age, BMI, alcohol intake, smoking status, diabetes and proton pump inhibitors use (by MaAsLin), only one taxon remained statistically significant between the two groups (Table 3). The relative abundance of *Haemophilus* was 100 times higher in sAH patients than in CAP patients (*p corr* = 0.002). We were able to trace 85% of the *Haemophilus* reads to a single species: *Haemophilus parainfluenzae*.

**Table 3:** Differences in the intestinal microbota between chronic alcoholic pancreatitis (CAP) and severe alcoholic hepatitis (sAH) at the genus level.

OTU	Family	*Genus*	Increased in	Fold increase	Relative abundance		Mann-Whitney (ACP vs sAH)		LEfSe (ACP vs sAH)		MaAsLin (ACP vs sAH)**	
Phyla					ACP	sAH	p	p corr	LDA	p	p	p corr
**Proteobacteria**	**Pasteurellaceae**	***Haemophilus***	**sAH**	**106.62**	**0.00005**	**0.00508**	**<0.01**	**0.00**	2.74	**<0.01**	**<0.01**	**<0.01**
	Enterobacteriaceae	Other[Klebsiella pneumoniae]*	ACP	2.06	0.00777	0.00377	0.04	0.16	2.69	0.04		
		*Serratia*	ACP	10.18	0.00056	0.00005	0.05	0.18				
	Moraxellaceae	*Acinetobacter*	ACP	1322.02	0.01322	0	0.01	0.12	3.14	0.01		
	Pseudomonadaceae	*Pseudomonas*	ACP	402.98	0.00403	0	0.05	0.18				
	Other	Other[Klebsiella pneumoniae]*	ACP	23.28	0.01535	0.00066	0.02	0.13	3.14	0.02		
	Alcaligenaceae	*Sutterella*	sAH	5.06	0.00148	0.00747	<0.01	0.01	2.78	<0.01		
	Comamonadaceae	[Aquabacterium parvum]*	ACP	62.50	0.00063	0	<0.01	0.05	2.64	<0.01		
	Campylobacteraceae	*Campylobacter*	sAH	51.65	0	0.00052	0.02	0.12	2.06	0.02		
Firmicutes	Lactobacillaceae	*Lactobacillus*	sAH	21.99	0.00565	0.12424	<0.01	0.01	4.04	<0.01		
	Streptococcaceae	*Lactococcus*	sAH	8.47	0.00010	0.00086	0.04	0.17	2.23	0.04		
	Enterococcaceae	*Enterococcus*	ACP	17.80	0.07650	0.00430	0.01	0.12	3.82	0.01		
	Lachnospiraceae	*Lachnospira*	sAH	15.56	0.00046	0.00713	<0.01	0.03	2.65	<0.01		
		*Anaerostipes*	sAH	5.71	0.00013	0.00071	0.02	0.12	2.16	0.01		
		*Roseburia*	sAH	1.76	0.00487	0.00857	0.02	0.13	2.70	0.02		
		*Epulopiscium*	ACP	418.45	0.00418	0	0.03	0.15	2.77	0.03		
	Ruminococcaceae	*Anaerotruncus*	ACP	2.35	0.00067	0.00029	0.01	0.12	2.03	0.01		
		*Faecalibacterium*	sAH	2.23	0.01867	0.04156	0.02	0.12	3.34	0.02		
		Other[Ruminococcus albus]*	sAH	9.02	0.00053	0.00478	0.04	0.16	2.64	0.04		
	Clostridiaceae	*Clostridium*	sAH	1.89	0.00406	0.00767	0.03	0.15	2.58	0.03		
		*SMB53*	ACP	98.21	0.00098	0	0.03	0.15	2.28	0.03		
	Veillonellaceae	*Dialister*	sAH	12.73	0.00050	0.00636	0.02	0.13	2.74	0.02		
Bacteroidetes	Flavobacteriaceae	*Flavobacterium*	ACP	16.07	0.00016	0	0.02	0.13	2.46	0.02		
	Prevotellaceae	*Prevotella*	sAH	3.04	0.01709	0.05193	0.03	0.15	3.53	0.03		
	[Paraprevotellaceae]	*Paraprevotella*	sAH	6.66	0.00014	0.00091	0.05	0.18				
	S24-7	[Muribaculum intestinale]*	sAH	12.41	0.00036	0.00451	0.03	0.15	2.61	0.04		
Actinobacteria	Coriobacteriaceae	*Atopobium*	sAH	7.95	0.00008	0.00062	0.01	0.12	2.20	0.01		
		[Raoultibacter massiliensis]*	ACP	8.63	0.00512	0.00059	0.01	0.12	2.76	0.02		
Fusobacteria	Fusobacteriaceae	*Fusobacterium*	sAH	5.94	0.00139	0.00824	0.05	0.18	2.89	0.04		

^*^Operational Taxonomic Unit (OTU) identified using BLASTN program (vBLAST + 2.6.0) from NCBI Blast against the NCBI 16 s Microbial database. Only hits with an overall sequence identity of 97% or more were considered.

^**^Covariates: age, sex, alcohol intake, smoking status, BMI, diabetes and proton pump inhibitors use.

ACP: alcoholic chronic pancreatitis, sAH: severe alcoholic hepatitis, LEfSe: LDA Effect Size, MaAsLin: Multivariate Association with Linear Models. In bold taxa with a p corr < 0.2 in MaAsLin.

### Differences in intestinal microbiota profile between alcoholic patients without complications and patients with severe alcoholic hepatitis

Principal coordinate analysis (PCoA) of unweighted (Supplemental Fig. 2A) distances revealed two clusters, corresponding to the two groups of patients (*p corr* = 0.03). There was a trend towards a difference in the weighted distances (*p*  corr = 0.07, Supplemental Fig. 2B). These results suggest a different composition (unweighted distances) and a trend towards a different structure (weighted distances) of the intestinal microbiota between the two groups. When comparing the specific taxa, differences between sAH patients and AC (*p* < 0.05, p corr < 0.20) were observed for 31 genera. LEfSe analysis revealed 23 taxa that were differed between the two groups (10 were more abundant in sAH patients and 13 were more abundant in AC, Supplementary Fig. 2C).

### Specific differences in taxa between groups

**Figure 4:**
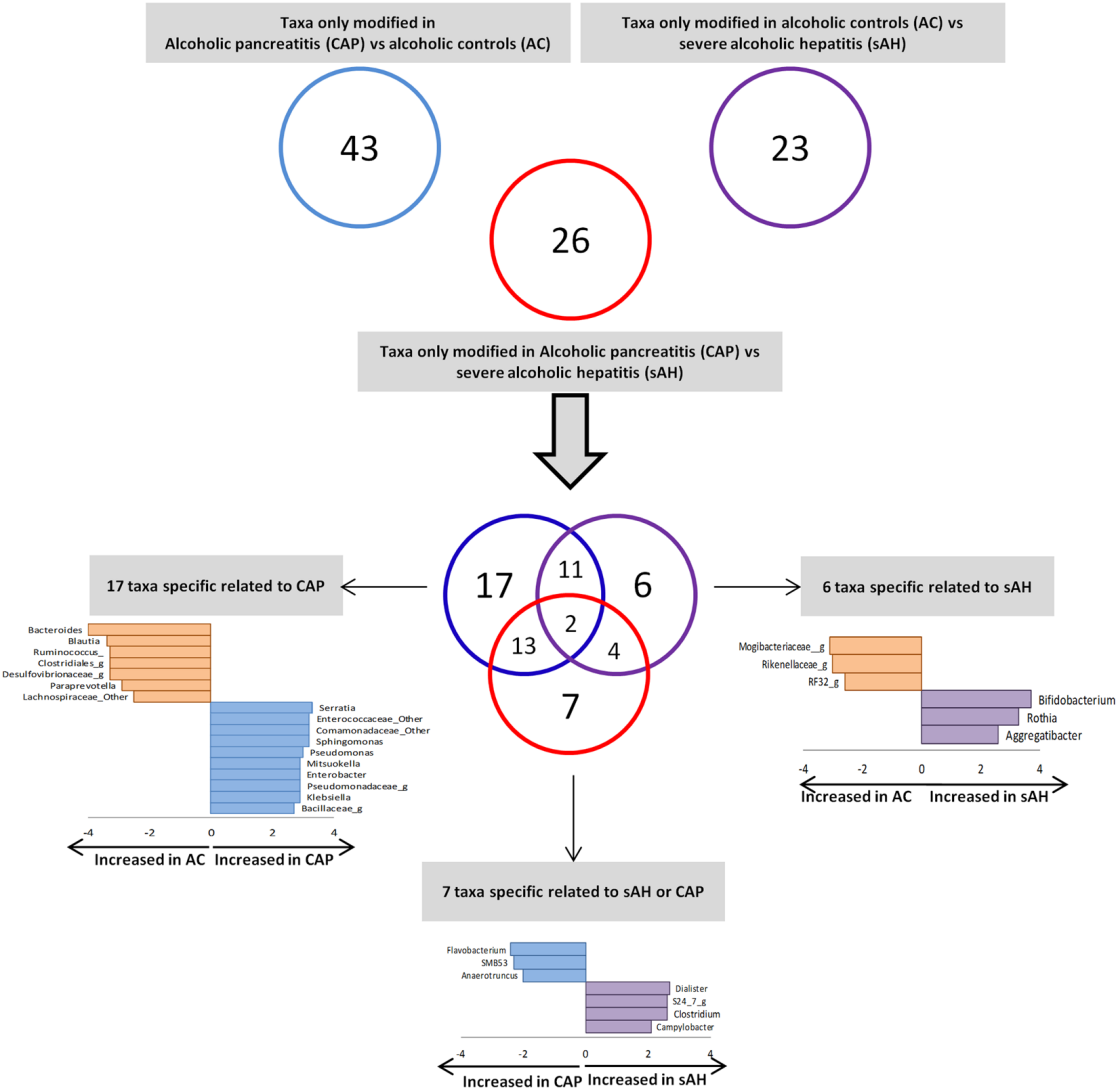
Venn diagram for the significant taxa (genus level) differing in all three analyses (CAP *vs*. AC, CAP *vs*. sAH and AC *vs*. sAH), showing the taxa displaying modifications specific to a particular complication (LDA score >2 and a *p* < 0.05 determined in a Wilcoxon signed rank test). CAP, chronic alcoholic pancreatitis; AC, alcoholic controls; sAH, severe alcoholic hepatitis.

When analyzing the taxa that were different between the three groups (in LEfSe, CAP vs. AC, sAH vs. AC and sAH vs. CAP), seven taxa differed only for comparisons of sAH and CAP, 17 differed in comparisons of CAP and AC and six differed in comparisons of sAH and AC. (Fig. 4). For taxa displaying differences only between CAP and sAH, the abundances of *Favobacterium, SMB53* and *Anaerotruncus* were higher in CAP patients and those of *Dialister, Clostridium, Campylobacter* and a member of the S24–7 family were higher in sAH patients, suggesting an effect independent of alcohol consumption on the development of theses complications.

## Discussion

In the present study, we analyzed the intestinal microbiota associated with CAP and its specificity. As alcohol intake alters intestinal microbiota composition^7^, we used a group of alcoholic patients without alcohol-related organ complications as the control group. Furthermore, as patients with excessive alcohol consumption may develop different complications, we investigated the intestinal microbiota in patients with CAP and with alcoholic cirrhosis presenting a sAH. We show that the intestinal microbiota associated with CAP has a different structure and composition as compared to AC and we identified several members of the intestinal microbiota as significantly associated with CAP. We also observed several differences in the bacterial composition of the intestinal microbiota between patients with CAP and sAH.

CAP patients had a lower microbial diversity than AC, whereas microbial diversity did not differ between CAP *vs*. sAH and AC *vs*. sAH patients. High bacterial diversity is usually associated with a healthy state and is thought to be crucial for the maintenance of immune system homeostasis^15^. A lower alpha diversity in patients with chronic pancreatitis has been reported before but only 25% of the patients included had alcoholic pancreatitis^12^. Moreover, blockade of the acinar cell exocytose of antimicrobial peptides in mice is associated with intestinal dysbiosis, inflammation and systemic bacterial translocation; this suggests that the secretion of antimicrobial peptides by pancreatic acinar cells regulates intestinal microbiota composition^16^. In AH, antimicrobial peptide levels are also low and are dependent on intestinal microbiota composition. Moreover, intestinal microbiota manipulation by fecal microbiota transplantation or prebiotics administration, prevents the decrease in antimicrobial peptide levels in the intestine and has hepatoprotective effects^6^. Overall, our results suggest that the decrease in intestinal microbiota richness and diversity may be due to the low antimicrobial peptide levels and exocrine pancreatic insufficiency observed in chronic pancreatitis.

The relative abundances of several potential pathogenic taxa *(Klebsiella, Enterococcus, Pseudomonas)* associated with systemic inflammations and infection^17^ were higher in CAP patients than in AC.

Previous studies have suggested that some bacteria may be involved in the pathogenesis of pancreatic diseases. *Granulicatella adiacens* and *Streptococcus mitis* from the oral microbiota have been shown to be associated with chronic pancreatitis^13^ while *Helicobacter pylori* infection has been shown to be associated with autoimmune pancreatitis^18^. Microorganisms may infect the pancreas through ascending gastric infections or retrograde transfer from the small intestine^19^. The intestinal dysbiosis observed in alcoholic patients was associated with an increase in intestinal permeability that facilitates the translocation of intestinal microbiota components and contributes to liver injury^20^. Moreover, a fluorescence *in situ* hybridization (FISH) study in the pancreatic duct biopsy specimens of patients with pancreatitis revealed the presence of a bacterial biofilm, including members of the Enterobacteriaceae family^21^.

We found that CAP patients had a higher relative abundance of *Enterococcus*. In a study that investigated intestinal microbiota using qPCR, *Enterococcus* was increased in patients with acute pancreatitis and positively correlated to plasma endotoxin levels^22^. *E. faecalis* is associated with impaired intestinal permeability via its gelatinase that alters the epithelial barrier and contributes to intestinal inflammation^23^. This could facilitate bacterial translocation and bile colonization, which may then come into contact with the pancreas. Conversely, *Enterococci* produce antimicrobial compounds^24^. This may account for decreased bacterial richness in CAP patients observed here, and the increases in *Enterococcus, Klebsiella, Pseudomonas* and *Serratia*, which are pathogenic in humans.

*Haemophilus parainfluenzae* (Pasteurellaceae family) abundance was increased in sAH patients. This commensal bacteria may also act as an opportunistic pathogen, causing invasive infections. Moreover, higher abundance of Pasteurellaceae was associated with acute-on-chronic liver failure and were found also to be an independent predictor of mortality in these patients^25^.

The dysbiosis observed in CAP was associated with a lower abundance of *Faecalibacterium* in CAP as compared to both AC and sAH patients. A decrease in the abundance of *Faecalibacterium prausnitzii* has also been reported in patients with chronic pancreatitis^12^, suggesting that this difference may be related to the pancreatitis itself rather than its cause*. F. prausnitzii* has anti-inflammatory activity^26^ and stimulates mucin and tight-junction protein synthesis, thereby improving intestinal barrier function^27^.

Interestingly, CAP patients also had lower levels of *Lactobacillus* than sAH patients. The abundances of *Lactobacillus* species are low in humans with alcoholic cirrhosis and in animal models of this disease^28^ and they can attenuate the features of alcoholic liver disease^29^. Lactobacilli secrete lactic acid, which reduces intestinal pH, the growth of commensal microbiota^30^ and the amounts of luminal bacterial products passing into the systemic circulation. Based on our results, we hypothesize that the lower levels of *Lactobacillus* in CAP patients than in sAH patients may underlie the higher abundances of pathogenic bacteria, such as *Pseudomonas, Klebsiella and Serratia*. However, Lactobacilli use in severe acute pancreatitis patients did not reduce the risk of infectious complications and was associated with an increase risk of mortality^31^.

In our study, there were less proton pump inhibitors users among patients with CAP as compared to the other two groups. This could have an impact on the intestinal microbiota as their use was associated with a decrease in the Bacteroidetes and an increase in Firmicutes at the phylum level and an increase in *Holdemania filiformis* and decrease in *Pseudoflavonifractor capillosus* at the species level^32^. Therefore, to take into account these differences, we adjusted for this potential confounding factor in the MaASLin model. Thus the changes observed in our populations were independent of the use of proton-pump inhibitors.

Our study has several limitations. Firstly, the cross-sectional design of the study precludes investigations of the underlying mechanisms and of the time-sequence of the association. Secondly, we lack an external validation cohort that could confirm our results. Nevertheless, we clearly demonstrate that, despite a similar amount of alcohol consumption, a specific intestinal microbiota is associated to the nature of alcohol-induced complication. Finally, the low power of this study, due to the small number of patients included, may also account for the smaller number of genera identified as differing between groups in MaAsLin than in the other analyses. However, there is no method to estimate a sample size for microbiota studies.

In conclusion, a specific intestinal microbiota signature is associated with the nature of the complication in alcoholic patients. However, it remains unclear whether CAP dysbiois is the cause or simply a consequence of pancreatitis. Further studies are required, with a longitudinal design, in which the microbiota of alcoholic patients can be assessed regularly and correlations with outcome can be assessed, together with studies in humanized animal models. Moreover, the specific microbial signature could be useful in identifying patients at risk to develop alcohol related complications and thus improve the management of these patients. These results are a key first step towards understanding why only 5% of alcoholic patients develop alcoholism-related pancreatic disorders.

## Methods

### Patients

For this prospective study, patients admitted in two tertiary hospitals for CAP or the management of excessive drinking were included in this study. Alcoholic patients were eligible for inclusion if they were between 17 and 75 years old and had been consuming at least 60 g of alcohol/day for more than five years, and were negative for hepatitis B surface antigens, hepatitis C or any other cause of liver disease. The exclusion criteria were gastrointestinal bleeding, bacterial infection, hepatocellular carcinoma, any other carcinoma, other severe associated disease, presence of anti-HIV antibodies, antibiotic intake in the last three months and refusal to undergo liver biopsy if required (abnormal liver function).

Three groups of patients were recruited:

- CAP patients (*n* = 24, recruited at Beaujon University Hospital, Clichy, France) admitted for an acute bout of pancreatitis or chronic pain. The additional inclusion criteria for this group were: 1) patients with CAP admitted for an acute bout of pancreatitis or chronic pain; 2) no other cause of chronic pancreatitis identified, despite an exhaustive search; 3) absence of pancreatic or extra-pancreatic tumors and 4) absence of liver disease. CAP was diagnosed on the basis of the presence of at least one of the following: pancreatic calcifications visible on CT scan or endoscopic ultrasonography; moderate-to-severe pancreatic ductal lesions on endoscopic retrograde or magnetic resonance pancreatography (Cambridge classification)^33^; histological features typical of pancreatitis on an adequate surgical specimen of the pancreas or recurrent acute alcoholic pancreatitis. Recurrent acute pancreatitis was defined by more than two bouts of acute abdominal pain with increases in serum pancreatic enzyme levels to more than three times the upper limit of the normal range.
- Alcoholic controls (AC, *n* = 45, recruited at Antoine-Béclère University Hospital, Clamart, France) admitted for the management of excessive drinking. The additional inclusion criteria for this group were: 1) admission for the management of excessive drinking, 2) no signs of acute pancreatitis or CAP (based on symptoms and morphological features), 3) no signs of liver disease (as described previously^5^).
- Alcoholic patients with severe alcoholic hepatitis and alcoholic cirrhosis (sAH, *n* = 13, recruited at the Antoine-Béclère University Hospital, Clamart, France) as defined by a histological score for alcoholic hepatitis ≥ 6 and neutrophilic infiltration on a liver biopsy^34^. The additional inclusion criteria for this group were: 1) admission for the management of excessive drinking, 2) no signs of acute pancreatitis or CAP (based on symptoms and morphological features). Feces collection was done before any steroid therapy.

General demographic and clinical characteristics were recorded for all patients at inclusion. Also a standardized questionnaire was used to collect information about alcohol consumption^35^.

We choose to compare the patients with acute bouts of CAP or pain exacerbation to sAH patients with alcoholic cirrhosis in order to have 2 groups of patients with an acute manifestation of an underlying chronic condition.

This study received IRB approval from the Ile de France VII ethics committee (Bicêtre Hospital, 94270 le Kremlin-Bicêtre, France). All methods were performed in accordance with the relevant local guidelines and regulations and all patients provided written informed consent for participation in the study.

### Collection of feces

Feces from the first bowel movement after admission were collected. An anaerobiosis generator (Anaerocult, Merck, Darmstadt, Germany) was used to favor the preservation of anaerobic bacteria. The samples were processed within 24 h and frozen for bacterial preservation at −80 °C and DNA extraction, as previously described^6^. Briefely, Bacterial DNA was obtained by homogenizing cecal content in a Guanidinium thiocyanate containing lysis buffer using a Fast Prep homogenizer. High quality bacterial DNA was extracted by successive steps of purification and precipitation using “Laboratory-made” buffers^36^. PCR were performed to prepare amplicons using V3-V4 oligonucleotides (PCR1F_460: 5′CTTTCCCTACACGACGCTCTTCCGATCTACGGRAGGCAGCAG3′, PCR1R_460: 5′GGAGTTCAGACGTGTGCTCTTCCGATCTTACCAGGGTATCTAATCCT3′). Amplicon quality was verified by gel electrophoresis and they were sent to the GenoToul plateform (Toulouse, France) for sequencing. All the samples were collected and processed in the same way.

### Analysis of the intestinal microbiota by 16s ribosomal RNA sequencing

The composition of the intestinal microbiota was analyzed by high-throughput sequencing with Illumina MiSeq technology, targeting the 16S ribosomal DNA V3-V4 region. Data was analyzed using QIIME v1.9.0. Paired-end reads were assembled with PANDAseq v 2.7, to generate the sequence of a 450-base pair amplicon^37^. Reads were demultiplexed and processed with the quantitative insights into microbial ecology (QIIME v1.9.0) pipeline, using its default parameters^38^. Chimeric sequences were identified *de novo*, on the basis of references, and were removed with usearch61^39^. The non-chimeric sequences were then clustered into operational taxonomic units (OTUs) displaying at least 97.0% sequence similarity, by a closed reference-based picking approach in UCLUST software applied to the Greengenes 13.8 database of bacterial 16S rDNA sequences^40^. The mean number of quality-controlled reads was 26008 ± 8106.69 (mean ± SD) per sample (minimum count: 8370.0, maximum count: 79884.0). After rarefaction at 7,000 reads per sample, bacterial alpha diversity was estimated on the basis of the Shannon’s index. OTUs with a prevalence <5% were removed from the analysis.

We assessed beta diversity with weighted and unweighted UniFrac distances. The weighted Unifrac metric is weighted by the difference in the abundance of OTUs from each community, whereas unweighted UniFrac considers only the absence/presence of OTUs, providing different information. Both are phylogenetic beta diversity metrics.

We investigated the OTUs not identified by QIIME further, using the BLASTN program (vBLAST + 2.6.0) from NCBI Blast, against the NCBI 16 s Microbial database. Only hits with an overall sequence identity of at least 97% were considered.

### Statistical analyses

The results are expressed as means ± SEM. Alpha diversity comparisons were performed with nonparametric Student’s *t*-tests and Monte Carlo permutations in QIIME.

Taxa were compared using Mann–Whitney U-tests and Adonis test was used to compare distance matrices in QIIME. Benjamini–Hochberg false discovery rate (FDR) correction (p corr) was used to correct for multiple hypothesis testing, when applicable. A FDR corrected p value < 0.2 was considered as statistical significant in these comparisons due to the exploratory design of the study.

LDA effect size (LEfSe) analysis was performed to identify the taxa displaying the largest differences in abundance in the microbiota between groups^41^. Only taxa with an LDA score >2 and a significance of α < 0.05, as determined in Wilcoxon signed-rank tests, are shown. The sizes of the circles in the cladogram plot are proportional to bacterial abundance.

MaAsLin (multivariate analysis by linear models) was used to identify associations between clinical metadata and microbial community abundance or function^42^. Sex, age, BMI, alcohol intake, smoking status, diabetes and proton pump intake were included as covariates in the MaAsLin. Relative abundances were subjected to arcsine root transformation before regression analyses using MaAsLin. Both LEfSe and MaAsLin were accessed online (http://huttenhower.sph.harvard.edu/galaxy/). For the MaAsLin compariason, a corrected p value < 0.2 (p corr) was considerd statisicaly significant.

The remaining comparisons were performed with R software v2.14.1. Unpaired *t*-tests or Mann–Whitney U-tests were used to compare continuous data between groups, according to data distribution. Chi^2^ or Fisher’s exact tests were used to compare discrete parameters between groups. A *p*-value < 0.05 was considered to be statistically significant, unless specified.

## Electronic supplementary material

Supplementary Data
