# Supplementary material for: Characterization of intestinal microbiota in alcoholic patients with and without alcoholic hepatitis or chronic alcoholic pancreatitis
Source: Sci Rep. 2018 Mar 19;8:4822. doi: 10.1038/s41598-018-23146-3 (PMC5859299; doi:10.1038/s41598-018-23146-3)
Supplement: Supplementary file 1 — Supplementary Data [file 41598_2018_23146_MOESM1_ESM.pdf]

Characterization of intestinal microbiota in alcoholic patients with and without alcoholic hepatitis or chronic alcoholic pancreatitis

Short Title **: Intestinal microbiota in pancreatitis**

Dragos Ciocan[1,2], Vinciane Rebours[3,4], Cosmin Sebastian Voican[1,2,5], Laura Wrzosek[1,2], Virginie Puchois[1], Anne-Marie Cassard[1,2], Gabriel Perlemuter[1,2,5]

[1]INSERM UMRS U996 - Inflammation, Cytokines and Immunopathology, DHU Hepatinov, F-92140 Clamart, France

[2]Univ Paris-Sud/Paris Saclay,F-92140, Clamart, France

[3]INSERM UMR 1149, DHU UNITY, CRI, University Paris 7, Paris, France.

[4]AP-HP, Pancreatology Department, Beaujon Hospital, Clichy, France.

[5]AP-HP, Hepatogastroenterology and Nutrition, Hôpital Antoine-Béclère, Clamart, France.

Correspondence: Prof. Gabriel Perlemuter, Service d'Hépato-Gastroentérologie et Nutrition, Hôpital Antoine-Béclère, 157 rue de la Porte de Trivaux, Clamart cedex F-92141, France; gabriel.perlemuter@aphp.fr.

Supplementary Data

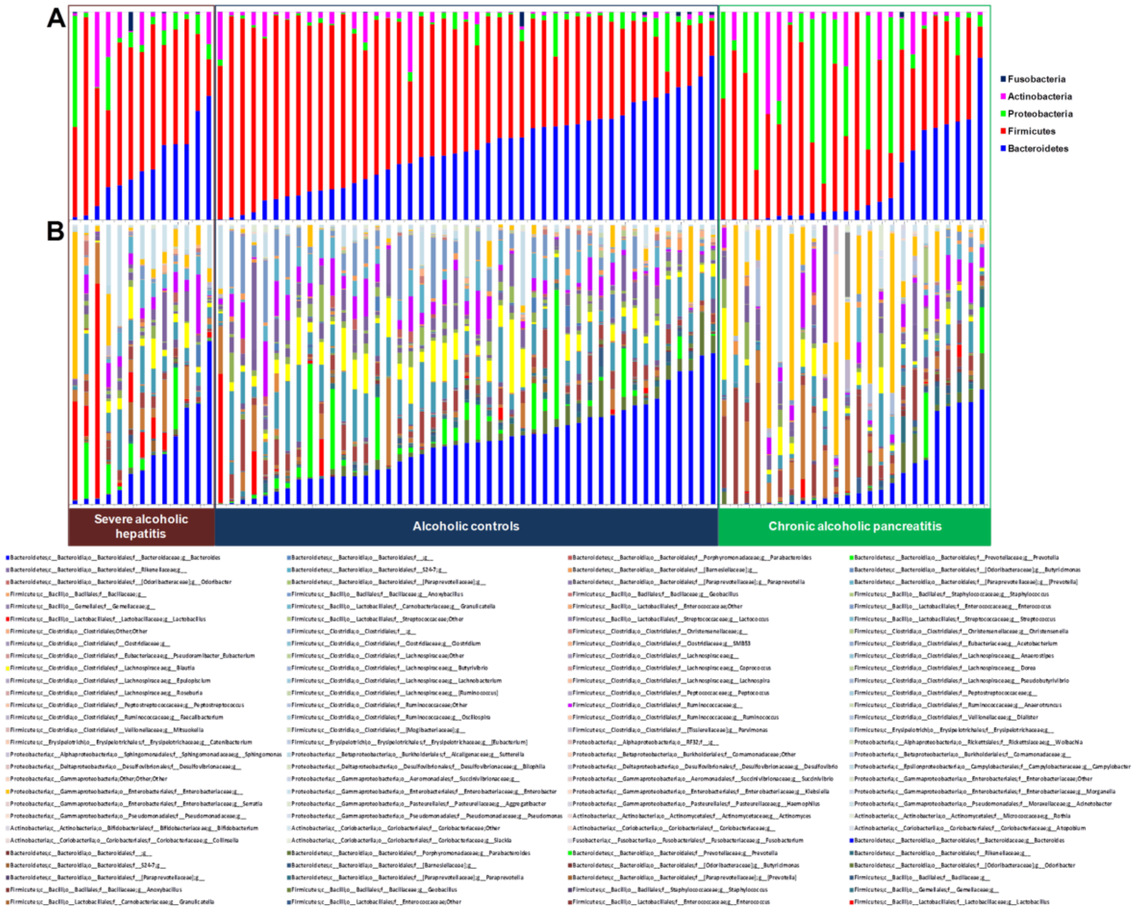

Supplementary Figure 1. Overall intestinal microbiota composition in alcoholic patients without (alcoholic controls) and with chronic alcoholic pancreatitis or severe alcoholic hepatitis at the phyla level **(A)** and at the genus level **(B).**

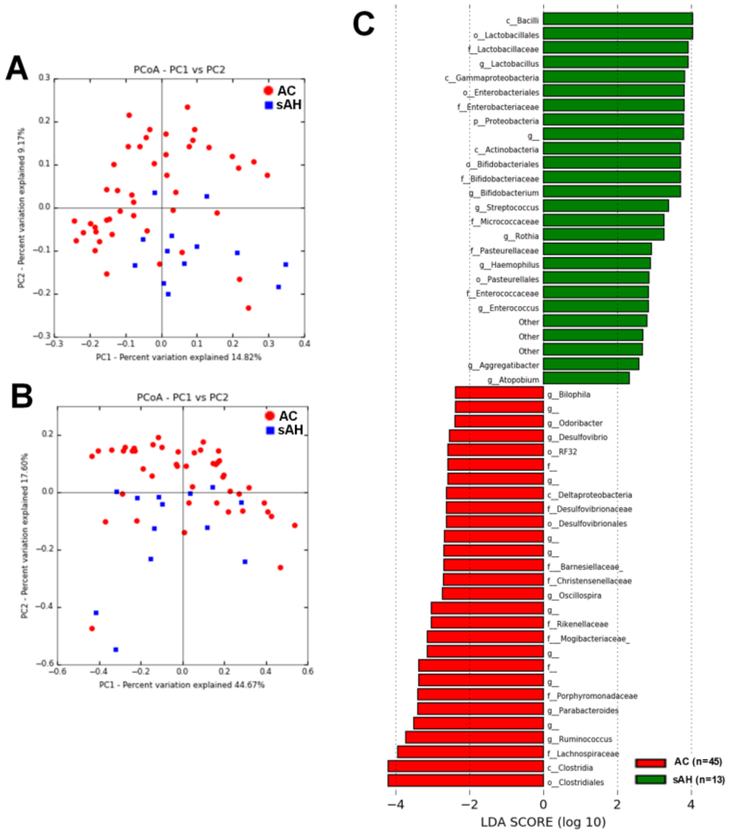

Supplementary Figure 2. Differences in the intestinal microbiota between alcoholic controls (AC) and patients with severe alcoholic hepatitis (sAH). (A) Unweighted UniFrac distances (quantitative method) showing differences in the structure of the intestinal microbiota between the two groups (p= 0.03, ANOSIM test); (B) Weighted UniFrac distances (qualitative method) showing no differences in the composition of the intestinal microbiota (p>0.05, ANOSIM test). Each point represents a subject and the distance between the points is proportional to the similarity in the intestinal microbiota. (C) LDA score of the taxa displaying differences in abundance between ACs and sAH patients. Taxa displaying enrichment in the microbiota of ACs (red) and sAH patients (green) are indicated by positive and negative LDA scores, respectively (taxa with a LDA score > 2 and a p<0.05 in a Wilcoxon signed rank test).
